# Conceptualizing Usability for the eHealth Context: Content Analysis of Usability Problems of eHealth Applications

**DOI:** 10.2196/18198

**Published:** 2021-07-27

**Authors:** Marijke Broekhuis, Lex van Velsen, Linda Peute, Meilani Halim, Hermie Hermens

**Affiliations:** 1 Roessingh Research and Development Enschede Netherlands; 2 Biomedical Signals and Systems Faculty of Electrical Engineering, Mathematics and Computer Science (EEMCS) University of Twente Enschede Netherlands; 3 Center for Human Factor Engineering of Health Information technology Department of Medical Informatics Amsterdam UMC, University of Amsterdam Amsterdam Netherlands; 4 Communication Sciences Faculty of Behavioural, Management and Social Sciences (BMS) University of Twente Enschede Netherlands

**Keywords:** usability benchmarking, eHealth systems, content analysis, usability framework, summative evaluation, mobile phone

## Abstract

**Background:**

Usability tests can be either formative (where the aim is to detect usability problems) or summative (where the aim is to benchmark usability). There are ample formative methods that consider user characteristics and contexts (ie, cognitive walkthroughs, interviews, and verbal protocols). This is especially valuable for eHealth applications, as health conditions can influence user-system interactions. However, most summative usability tests do not consider eHealth-specific factors that could potentially affect the usability of a system. One of the reasons for this is the lack of fine-grained frameworks or models of usability factors that are unique to the eHealth domain.

**Objective:**

In this study, we aim to develop an ontology of usability problems, specifically for eHealth applications, with patients as primary end users.

**Methods:**

We analyzed 8 data sets containing the results of 8 formative usability tests for eHealth applications. These data sets contained 400 usability problems that could be used for analysis. Both inductive and deductive coding were used to create an ontology from 6 data sets, and 2 data sets were used to validate the framework by assessing the intercoder agreement.

**Results:**

We identified 8 main categories of usability factors, including basic system performance, task-technology fit, accessibility, interface design, navigation and structure, information and terminology, guidance and support, and satisfaction. These 8 categories contained a total of 21 factors: 14 general usability factors and 7 eHealth-specific factors. Cohen κ was calculated for 2 data sets on both the category and factor levels, and all Cohen κ values were between 0.62 and 0.67, which is acceptable. Descriptive analysis revealed that approximately 69.5% (278/400) of the usability problems can be considered as general usability factors and 30.5% (122/400) as eHealth-specific usability factors.

**Conclusions:**

Our ontology provides a detailed overview of the usability factors for eHealth applications. Current usability benchmarking instruments include only a subset of the factors that emerged from our study and are therefore not fully suited for summative evaluations of eHealth applications. Our findings support the development of new usability benchmarking tools for the eHealth domain.

## Introduction

### Background

Usability tests of eHealth applications can be either formative (where the aim is to detect usability problems) or summative (where the aim is to benchmark usability). Formative usability tests use qualitative methods, *think aloud* protocols [1,2], interviews [3], cognitive walkthrough [4], heuristic evaluation [5], or quantitative methods, such as user task performance [6]. Formative tests are mainly used to track usability problems, which are crucial for optimizing a system. However, they do not provide an absolute score of a system’s usability. Instead, this can be achieved via usability benchmarking methods during summative evaluations. A usability benchmark is a clear indicator of when the usability of an eHealth application is considered sufficient or insufficient. Furthermore, benchmarking makes it easy to compare the usability of an eHealth application with that of competitors, or to compare scores of new and old versions of the same system to determine whether usability has dropped, improved, or stayed the same. Benchmarking the usability of an eHealth application is most frequently done using questionnaires [7], such as the Poststudy System Usability Questionnaire (PSSUQ) [8], the questionnaire for user interface satisfaction [9], and the system usability scale (SUS) [10]. In addition, there are dedicated eHealth-specific usability benchmarking instruments, such as the Health Information Technology Usability Evaluation Scale (Health-ITUES) [11] and the Mental Health App Usability Questionnaire (MAUQ) [12]. The SUS is currently the most popular usability benchmarking tool for eHealth applications [13]. However, a recent examination of the suitability of the SUS to the eHealth context found that this instrument was not sufficient [14]. All of these questionnaires provide a verdict on usability based on the outcomes of the average scores of user-rated items. Each of these items is related to overarching factors that make up the construct of usability. Traditionally, usability is broken down into three factors: effectiveness, efficiency, and satisfaction [15]. However, each questionnaire proposes a different set of factors and thus, provides a different interpretation of usability. For example, the PSSUQ assesses usefulness, information quality, and interface quality, whereas the Health-ITUES measures the quality of work life, perceived usefulness, ease of use, and user control. Finally, the SUS has no constructs, only items that result in a single score for overall usability without defining what this score means. Thus, the proper benchmarking of usability should start by defining which factors make up the usability of a particular type of system [16].

It has been argued that usability should be considered from the perspective of the system domain [17]. eHealth applications are designed to inform about, prevent, diagnose, treat, or monitor health conditions. This requires users to, for example, understand the health information the system offers, need to be able to keep track of their progress, or need to be able to correctly perform exercises or fill out questionnaires based on the information that is available in the system. These activities can be complicated if patients have low health literacy [18] or if there are health impairments that are common for the intended patient group, which could hinder user-system interaction [19,20]. Furthermore, eHealth applications that are designed for a large audience, such as preventative healthy aging systems, need to consider an extremely diverse user group in terms of motivation and educational level [21].

The problems with current usability benchmarking tools for the eHealth context stem from a general lack of understanding of usability within the eHealth context [12]—eHealth usability. Many studies that attempt to classify usability factors for eHealth do so via a theoretical reclassification of earlier, traditional models [22-27]. This means that we merely rephrase or recategorize the same factors for eHealth instead of eliciting domain-specific usability factors. In order to gain insights into the factors that make up eHealth usability, we need to go back to the drawing board: analyzing problems end users experience when interacting with eHealth applications. The proper usability of eHealth applications is not just about smooth navigation, clear understanding of used language, or prevention of system errors but also involves the patient’s perspective and focuses on understanding how a system supports them in prevention, diagnosis, treatment, or monitoring of their health condition [28-30]. However, chronic illnesses can increase patients’ feelings of stress and anxiety [31], which can affect the manner in which they interact with an eHealth application and thus the perceived usability. In contrast, for health professionals, for example, nurses, proper usability could mean an entirely different thing. For them, it is important that the system fits within their daily work routine. The study by Ash [32] describes how digital patient care information systems, while implemented with good intentions to make work easier for health professionals, can have unforeseen negative consequences (eg, additional workload or information overload of overfragmentation of data), making it unusable for the intended user group. A thorough understanding of eHealth usability supports formative evaluation methods that aim to elicit lists of usability problems, as well as supporting benchmarking tools.

### Objectives

By analyzing multiple data sets of usability problems found in contemporary eHealth applications, we propose a conceptualization of usability for the eHealth domain from the patient’s perspective. An overview of eHealth-specific usability factors helps usability practitioners to link usability problems to an overarching classification that is tailored to the specific medical context in which these applications are embedded.

## Methods

Data sets of usability tests were collected to conduct a content analysis of usability problems found in eHealth usability tests.

### Data Source Collection

We analyzed 8 data sets from different usability tests conducted at institutions affiliated with the researchers. The data sets were strategically chosen to reflect a wide range of eHealth applications with different end-user groups, devices, and health goals. A data set was included if the eHealth application was recently developed; usability problems were elicited via at least one qualitative data collection method (eg, thinking aloud, interviews, and observations); and the participants of the usability tests consisted of patients.

The following eHealth applications were included in this study: (1) Stranded, a web-based gamification application in which users can progress in the game by regularly performing physiotherapeutic exercises that are scheduled by a physiotherapist [14]; (2) a web-based screening module provided by a tablet and a care robot (NAO, a humanoid robot from SoftBanks Robotics), in which older adults completed a frailty test and performed physical exercises [33]; (3) cVitals, a home-monitoring module for patients with chronic obstructive pulmonary disease to monitor their health, which consists of a web application that is connected to a blood pressure monitor and weight scale monitor; (4) Council of Coaches, a web-based multi-agent virtual coaching platform for older adults to support a healthy lifestyle via dialogues, web-based coaching, and exercises from multiple virtual coaches that represent various health dimensions (eg, social and physical and mental health); (5) Pandit, a web application for patients with diabetes that provides insulin dosing advice using a clinical decision support system [34]; (6) Pregnancy and Work application (in Dutch: Zwangerschap en Werk) a mobile app for pregnant women to inform them about the rules and regulations on the work floor with regard to pregnancy; (7) FatSecret, a mobile food diary app for diabetes patients; and (8) Hospitality app, a mobile app that provides valet navigation service for out-clinic patients to heighten hospitality toward patients and facilitate hospital attendance [35].

### Usability Problems and Severity

The data sets had a total of 486 usability problems. We excluded usability problems that had unclear formulation, were duplicated, or were unrelated to usability (eg, user experience and motivation). For example, the problem *User presses the home button of the iPad for too long, after which Siri comes up instead of home screen* (from data set 3) is a problem with the device (tablet) and not with the eHealth application. Another problem, *Not willing to watch the video and starts practicing* (from data set 2), is a problem with user motivation and not with the eHealth application. In addition, the problem, *It took users a long time to find the correct functions* (from data set 7) does not specify what functions are difficult to find. Finally, the problem *Does not like the music* (from data set 1) is not a usability problem but a user experience problem.

A total of 86 usability problems were eliminated from the data set, resulting in 400 usability issues that were suitable for the analyses. Each usability problem was assigned to a severity category. Most data sets included severity ratings based on the severity index of Duh et al [36]. This categorization differentiates among minor, serious, and critical usability problems. A minor usability problem occurs infrequently among the participants or the problem only increases the task completion time slightly. A serious usability problem frequently occurs among the participants or the problem severely increases the task completion time. A critical usability problem occurs when all participants have the same problem or the problem prevents participants from completing tasks. In case a data set consisted of different severity index, this index was transposed to the index of Duh et al [36].

Table 1 presents a complete overview of the characteristics of the eHealth applications, the end-user group, and the evaluation method per system.

**Table 1:** Overview of data sets (N=8).

Data set	eHealth application	Description of app	Main health goal	Device platform	Target end-user group	Participants, n	Evaluation method	Length of session (minutes)	Usability problems, n
1	Stranded	Web-based gamified app	Offers fall prevention training via video instructions in a gamified environment	Computer	Prefrail^a^ and frail older adults (aged ≥65 years)	19	Concurrent think aloud and screen capture recordings	60	66
2	N/A^b^	Web-based screening module	Identifies frailty levels among older adults and supports physical exercising	Tablet and social robot	Prefrail and frail older adults (aged ≥70 years)	20	Video observation	50	64
3	cVitals	Home-monitoring tool	Allows self-management of health by providing and supporting health measurements at home	Smartphone	Patients with heart failure or COPD^c^ (aged ≥65 years)	10	Concurrent think aloud and observations	60	39
4	Council of Coaches	Web-based coaching platform with virtual coaches	Supports a healthy lifestyle for older adults	Computer	Older adults (aged ≥55 years)	18	Think aloud and observations	60	60
5	Pandit	Web-based application	Allows self-management of health by providing insulin dosing advice	Computer	Patients with type 2 diabetes (aged 40-64 years)	5	Concurrent think aloud and observations	15	28
6	Pregnancy and Work	Informational application	Provides information on health risks and regulations during pregnancy	Smartphone	Pregnant women (aged 25-40 years)	12	Concurrent think aloud and observations	45	84
7	FatSecret	Calorie counter application	Provides nutritional information	Smartphone	Older adults with type 2 diabetes (aged ≥55 years)	10	Concurrent think aloud and observations	15	41
8	Hospitality app	Patient hospitality app	Provides information on how to prepare for a visit to medical facilities	Smartphone	Prefrail and frail older adults (aged ≥65 years)	8	Concurrent think aloud and observations	30	18

^a^Prefrail refers to the initial state of a health condition called *frailty*. This condition entails a gradual decline in physical and cognitive functions, mostly occurring among older adults, that can lead to recurrent falls, hospitalization, and even death [37].

^b^N/A: not applicable.

^c^COPD: chronic obstructive pulmonary disease.

### Data Analysis

A content analysis was conducted according to the methods of Bengtsson [38], which consists of four stages: decontextualization, recontextualization, categorization, and compilation. Below, we describe the process for each phase. The content analysis was performed by 3 people, all with a background in behavioral sciences, but with different degrees of expertise in coding qualitative data, namely novice (MH), experienced (MB), and expert (LVV).

First, in the decontextualization phase, 2 researchers (MB and MH) familiarized themselves with the data sets. Then, they independently started an inductive coding process. Each usability problem was assigned a code that represents the usability factor. On the basis of data sets 1, 2, and 3, each researcher developed their own codebook. These two codebooks were discussed and merged in one mutually agreed upon codebook, consisting of 9 main categories and 32 factors. Second, in the recontextualization phase, 2 researchers (MB and MH) independently recoded data sets 1-3 using the new codebook. If they found a usability problem that they could not classify using the codebook, a new code was added to the codebook. The resulting codebooks were then compared and discussed, leading to an updated codebook. These steps were performed several times until no new codes emerged. Third, in the categorization phase, definitions for each factor in the updated codebook were formulated, which now consisted of 10 categories and 28 factors. Then, a third independent researcher (LVV) familiarized himself with the data, codebook, and definitions. On the basis of triangular findings, alterations were made to the codebook, resulting in 9 categories and 24 factors. Finally, in the compilation phase, data sets 4, 5, and 6 were independently recoded by two researchers (MB and LVV) using the codebook (deductive coding). Discussions revealed that, although no new categories or factors emerged, there was some overlap in the definitions of some categories and factors that caused confusion about which factor to assign to the usability problem. Therefore, the codebook and definitions were adjusted. The final codebook consisted of 8 categories and 22 factors. The intercoder agreement between researchers MB and LVV was determined by coding data sets 7 and 8 and calculating Cohen κ values for both the category and variable levels.

Cohen κ is the most widely used means for measuring the intercoder agreement. However, it has its limitations, especially for nondichotomous variables, a measure of relative rather than absolute agreement [39]. One of the main problems with Cohen κ is that the higher the number of categories, the less likely there is chance for strong intercoder agreement when using the Cohen κ [40]. Therefore, we supplemented Cohen κ with a percentage agreement. As a final part of the analysis, we compared the number of minor, serious, and critical usability problems between the usability factors and categories to analyze whether some factors or categories had a significantly higher number of severe usability problems than others.

## Results

### Intercoder Agreement

Validation of the analysis was performed by calculating Cohen κ values for both category and factor levels (Table 2). The resulting Cohen κ values were ≥0.62, both on usability category and factor levels; all percentages were ≥66%. These scores can be interpreted as sufficient agreement between the researchers [41].

**Table 2:** Intercoder agreement expressed as Cohen κ and percent agreement for usability categories and factors.

Data set			Agreement level		
			Usability category		Usability factor
**Data set 8**					
	Usability problems, n	18		18	
	Percent agreement (%)	72		67	
	Cohen κ	0.62		0.63	
**Data set 7**					
	Usability problems, n	41		41	
	Percent agreement (%)	76		66	
	Cohen κ	0.67		0.62	

### Usability Factors for eHealth Applications

#### Overview

The ontology for usability problems for eHealth applications, which resulted from the coding process, consists of 8 overarching usability categories and 21 factors (Table 3). We differentiated between general usability factors (ie, design clarity, interface organization, and navigation) and eHealth-specific usability factors (ie, fit between system and health goals, accommodation to physical limitations, and procedural health-related information). The difference between these 2 types of usability factors (general and eHealth-specific) is that general factors are factors found in eHealth applications that we considered not unique to the eHealth domain (eg, system errors could occur regardless of the type of system), whereas eHealth-specific usability factors are factors related to the medical context in which eHealth applications are embedded (eg, health information, medical terminology, and health goals).

**Table 3:** Ontology for usability problems in eHealth applications.

Category of usability problem and usability factor			Type of usability factor
**Basic system performance**			
	Technical performance	General	
	General system interaction	General	
**Task-technology fit**			
	Fit between system and context of use	General	
	Fit between system and user	General	
	Fit between system and health goals	eHealth-specific	
**Accessibility**			
	Accommodation to perceptual impairments or limitations	eHealth-specific	
	Accommodation to physical impairments or limitations	eHealth-specific	
	Accommodation to cognitive impairments or limitations	eHealth-specific	
**Interface design**			
	Design clarity	General	
	Symbols, icons, and buttons	General	
	Interface organization	General	
	Readability of texts	General	
**Navigation and structure**			
	Navigation	General	
	Structure	General	
**Information and terminology**			
	System information	General	
	Health-related information	eHealth-specific	
**Guidance and support**			
	Error management	General	
	Procedural system information	General	
	Procedural health-related information	eHealth-specific	
**Satisfaction**			
	Satisfaction with system	General	
	Satisfaction with system’s ability to support health goals	eHealth-specific	

#### Category 1: Basic System Performance

This category includes usability problems related to the system’s technical stability and the user-system interaction. The factor *technical performance* describes usability problems related to the technical performance of the system, such as system errors, response times, and compatibility with external devices. An example of such a usability problem is the connection with a blood pressure monitor (Omron and Withings) does not work (data set 3, usability problem number 32). The factor *general system interaction* includes usability problems related to general system interaction elements (eg, use of buttons, scroll bars, swipes, and clicks) and concepts (eg, *the types of data entry are inconsistent through the app: String and integer entry, choices, scrolling through dates* [data set 7, usability problem number 1]).

Technical problems, such as nonresponsive buttons, can negatively affect efficient system interaction and perceived ease of use. These system errors can seriously hinder task completion and influence users’ opinions of other usability aspects. For example, if page load time takes too long (data set 1, usability problem number 19), a user can also give low ratings to the system’s ease of use, navigation, or satisfaction. Good technical performance of the system is essential to facilitate smooth and easy user-system interaction.

#### Category 2: Task-Technology Fit

Usability problems found in this category address the match between the system on the one hand, and the user, their context, and health goals, on the other hand. As such, this category is related to the model of Goodhue and Thompson [42], which defines task-technology fit as “the degree to which a technology assists an individual in performing his or her portfolio of tasks.” The three factors describe usability problems that occur because the eHealth application is not considered suitable because of (1) the daily (clinical) context of use in which the app is to be implemented (eg, participant indicates that she could not print something from the phone easily [data set 6, usability problem number 86]), (2) the needs of the intended end-user group (eg, *the default given for date of birth might not be optimal from the perspective of the average diabetic* [data set 7, usability problem number 3]), and (3) the intended health goals the app is designed to support (eg, *the user did not take the system seriously, it was perceived more as a game than as a tool for living more healthily* [data set 4, usability problem number 12]). When users perceive a good match between the system and the context, health goals, and themselves, it will lead to not only a more positive impression of the usability of an eHealth application but also a better understanding of its added value.

#### Category 3: Accessibility

The category *accessibility* addresses usability problems that stem from the system’s inability to adequately consider or compensate for physical (eg, *not able to do the exercise completely due to physical impairments* [data set 2, usability problem number 15]), cognitive (eg, *the explanation in the support video in the mailbox goes too fast for the user* [data set 1, usability problem number 37]), or perceptual (eg, *not able to hear NAO due to hearing impairment* [data set 2, usability problem number 38]) limitations or impairments that are common to the identified patient groups. These impairments could affect how the user interacts with the system. Problems with moving one’s wrist, or having tremors, could make it more difficult to move a mouse and click on objects or buttons. The system could make the buttons larger to make it easier for patients to click on it. Cognitive problems, such as concentration or memory problems, could make a person more forgetful of the things he or she has read. The system can accommodate this by repeating information. To address perceptual problems, for example, bad vision, the system could make the font size larger, so that texts are easier to read.

We were aware that the category *accessibility*, as the name indicates, is strongly linked to the concept of accessibility [43,44] or related concepts such as universal design [45] and user-sensitive inclusive design [46]. Although it is generally argued that these three concepts are not part of the system usability, previous studies [43-46] have acknowledged that there is a strong link. Our decision to include the category of *accessibility* hinges on three arguments. First, accessibility, as part of universal access, can promote usability [45]. Second, although accessibility is considered a functional and objective prerequisite for systems, user evaluation of these functionalities remains subjective and from a user perspective, cannot be perceived as separate from the general usability of a system. Third, eHealth applications are often designed for specific patient groups who can have physical, cognitive, or perceptual impairments or limitations. The user-friendly design of such systems therefore inherently provides access to people with such disabilities.

#### Category 4: Interface Design

The fourth category, *interface design*, focuses on the visibility of general user interface (GUI) elements. It has four variables. The first variable, *design clarity*, includes usability problems related to the size and clarity of a single GUI element (eg, buttons, icons, and graphics). One of the problems we found was that *calendar (buttons) was too small, and the user accidentally tapped the field behind the calendar* (data set 6, usability problem number 13). The variable *symbols, buttons, or icons* covers usability problems about the purpose of the GUI elements in the system. Does the user understand what these are for? For example, *it is unclear what it means when the light of the Withings blood pressure monitor blinks* (data set 3, usability problem number 1). The third variable, *interface organization*, concerns the placement and organization of GUI elements on a single screen, for example, *the user had problems with the layout of the answering options with a 7 pt Likert scale* (data set 4, usability problem number 3). The last variable, *readability of texts*, describes usability problems related to ease (eg, format, organization, and information density) with which a user can read a text, as well as typographic aspects (eg, font size and line height). For example*, information overload in frequently asked question takes a long time to find answers* (data set 8, usability problem number 19).

#### Category 5: Navigation and Structure

This category describes usability problems related to the simplicity and intuitiveness with which a user can move between different system components and a general understanding of the different system components. The factor *navigation* relates to the flow between multiple pages and is able to make correct predictions of what can be found in the system. An example of a navigational problem is *that navigation with the game is unclear, and the user uses nongaming elements to navigate between the different screens* (data set 1, usability problem number 30). Good navigation allows for efficient user-system interaction, that is, it takes less time to complete tasks, and it is easily understood how to perform the tasks [47]. Although system structure is often mentioned as a basic concept that users should be able to understand while using a system [48,49], there is little clarity with regard to the meaning of this concept. In our analysis, the usability factor *structure* emerged as one that relates to the user’s understanding of the system components and the relationships between these different system components. An example of a structural issue is *the connection between the beachcomber cabin (for storing stranded items) and the drift bottles (for receiving stranded items) is unclear* (data set 1, usability problem number 59). A system structure in which users easily understand how different components relate to each other will positively affect the efficiency and effectiveness with which users can complete system and health-related tasks.

#### Category 6: Information and Terminology

This category consists of explanatory, nonaction-related system information and terminology in the app. Usability problems can include issues with understanding labels or terminology, the level of language, or the use of a foreign language. In this category, we made a distinction between system and health-related information. The first type includes information about the understandability of explanatory, nonaction-related information and terminology about the system, such as the use of nonnative language (eg, *chronic obstructive pulmonary disease questionnaire appears to be in English instead of Dutch* [data set 3, usability problem number 35]), whereas the latter includes information related to the understandability of explanatory, nonaction-related information about health, medical terminology, or achieving health goals (eg, *patient is not familiar with the word hypoglycemia [does not understand if this means a high or low blood sugar level], but he does understand hypo* [data set 5, usability problem number 18]). It is important for eHealth applications that are designed for patients to have medical terminology that is aligned with the patients’ vocabulary.

#### Category 7: Guidance and Support

The *guidance and support* category describes usability problems that occur when the system does not provide sufficient support and feedback for tasks that the user has to perform and (potential) errors the user makes. The variable *error management* refers to the (lack of) feedback mechanisms that are incorporated within the system to prevent user errors. For example, “It was not clear that an incorrect blood sugar level was entered, the error pop-up only explained that there was insufficient information related to the field fasting blood sugar levels” [data set 5, usability problem number 12]. The other two variables in this category covered procedural information. Ummelen [50] describes procedural information as information that is related to conditions for actions, the manner in which actions are to be performed, and results and feedback from these actions. Next, a distinction is made between procedural *system* information and procedural *health-related* information. The first describes problems related to system actions (eg, “The system does not explain that the age of the user should be entered numerically, not alphabetically” [data set 4, usability problem number 6]). The second type of procedural information describes problems related to health-related tasks, such as performing physical exercises, filling in food diaries, and completing health questionnaires to measure physiological parameters. For example, i*t is unclear that the first time is to watch how NAO [a social robot] does the exercise* (data set 2, usability problem number 44). These factors, such as error prevention and feedback, are embedded in general usability design principles and heuristics [51]. However, for eHealth applications, these factors are also important to support users in the self-management of their health. For example, being unable to correctly perform physical exercises or not knowing if an exercise has been finished can be detrimental to perceived usability. Users do not know how to successfully complete health tasks and thus, do not know whether and how these tasks contribute to their health.

#### Category 8: Satisfaction

This final category concerns the user’s satisfaction with the system and addresses the subjective opinion of the user on, or likeability of, an eHealth application. System satisfaction is one of the standard usability variables according to the ISO (International Organization for Standardization) definition [15] and includes usability problems related to the user’s satisfaction with the system in general. In addition to this factor, we have identified a second type of satisfaction, namely *satisfaction with the system’s ability to support health goals*. This second variable was added because although the user could believe that the system is nice or fun to use, this does not mean that the system also satisfactorily supports them in their intended health goals. The difference between these two variables is illustrated as follows: the users did not like it when different virtual coaches contradict one another (data set 4, usability problem number 20). This is a system-satisfaction problem. Some users also mentioned that they did not like the background stories of the virtual coaches (data set 4, usability problem number 15). This is a satisfaction problem related to the potential of the system to support health goals.

### Descriptive Analysis

The eHealth usability ontology includes a total of 21 usability factors, of which 7 are eHealth-specific and 14 are context-independent. Table 4 displays the distribution of 400 usability problems that were included in the analyzed data sets over the different factors. It shows that about 69.5% (278/400) of the identified issues were of a basic nature and 30.5% (122/400) were health specific. This distribution is also present when we focus on minor, serious, and critical usability problems.

Next, we determined the number of minor, serious, and critical usability problems across the 8 categories (Table 5). The guidance and support category contained the highest number of usability problems, followed by the interface design, basic system performance, and navigation and structure categories. Accessibility and satisfaction had the lowest number of usability problems. Interestingly, although the interface design category has a high number of usability problems, which is 24% (96/400) of the total number of usability problems, only 7 usability problems were marked as critical.

**Table 4:** Number of basic and health usability problems according to severity category.

Factor type	Usability problems (n=400), n (%)	Severity category, n (%)		
		Minor (n=186)	Serious (n=147)	Critical (n=67)
Basic	278 (69.5)	130 (69.9)	101 (68.7)	47 (70.1)
Health	122 (30.5)	56 (30.1)	46 (31.3)	20 (29.9)

**Table 5:** Number of usability problems of usability categories according to severity level.

Usability category	Severity category				Total (n=400), n (%)
	Minor usability problems (n=186), n (%)	Serious usability problems (n=147), n (%)	Critical usability problems (n=67), n (%)		
Basic system performance	32 (17.2)	10 (6.8)	14 (20.9)	56 (14)	
Task-technology fit	16 (8.6)	7 (4.8)	5 (7.5)	28 (7)	
Accessibility	2 (1.1)	5 (3.4)	1 (1.5)	8 (2)	
Interface design	51 (27.4)	38 (25.9)	7 (10.4)	96 (24)	
Navigation and structure	12 (6.4)	18 (12.2)	12 (17.9)	42 (10.5)	
Information and terminology	13 (6.9)	13 (8.8)	1 (1.5)	27 (6.7)	
Guidance and support	56 (30.1)	55 (37.4)	25 (37.3)	136 (34)	
Satisfaction	4 (2.1)	1 (0.7)	2 (3)	7 (1.7)	

## Discussion

### Principal Findings

On the basis of the results of this study, we can reconceptualize the traditional concept of usability in the eHealth context. Our analysis of usability problems in eHealth applications identified 8 main categories for eHealth usability: (1) basic system performance, (2) task-technology fit, (3) accessibility, (4) interface design, (5) navigation and structure, (6) information and terminology, (7) guidance and support, and (8) satisfaction. In each usability category, we made distinctions between factors that were related to general usability (basic usability factors) and those related to the health goals of the system, the medical context, or the characteristics of the intended patient group (health usability factors). We identified 14 general factors and 7 eHealth-specific factors from the analysis. Further examination of the number of usability problems between general and eHealth-specific usability factors revealed that 69.5% (238/400) of all usability problems were related to general factors and 30.5% (122/400) to eHealth-specific factors. When looking at the severity categories (minor, serious, and critical), we observed the same distribution (70:30) between these two types of factors. This implies that when one applies a general usability benchmarking instrument for evaluating eHealth applications, such as the SUS [10] or the PSSUQ [8], the final score cannot fully cover all usability problems (ie, eHealth-related ones), as eHealth-specific attributes of usability are not taken into account in these instruments. In other words, these general instruments can only explain a maximum of 70% of the app’s usability. To fully assess the usability of eHealth applications, it is necessary to consider these additional eHealth-specific factors.

### Comparison With Prior Work

The finding that the context, be it eGovernment, eCommerce, or eHealth affects usability is, of course, not surprising. Context has been a prominent factor in the definition of usability since the emergence of this construct [52]. However, no studies have yet identified the factors that comprise the usability construct in the eHealth context. In contrast, much research has been conducted to create generic instruments to obtain a rapid and very general assessment of the status of usability of systems, regardless of the system domain or context. Our results showed that the factors related to the medical context influence approximately 30.5% (122/400) of the usability problems that users encounter in eHealth applications, which is a substantial part. Interestingly, several usability evaluation studies of eHealth applications implicitly mentioned how the medical or health context affects the usability of these systems [53-55]. However, these health-related problems are often inadequately categorized under broad concepts, such as usefulness, ease of use, and layout. Our study ties together these findings by providing a fine-grained ontology to which all these health usability problems can be linked. This allows for a better understanding of the usability of eHealth applications. We have provided several examples found in recent literature of why this is necessary.

First, Voncken-Brewster et al [53] found that users, that is, people with a chronic illness, believed that the feedback of the system was not suitable for them because of the progressive physical limitations they experienced. In this study, they classified their usability problems into three main categories: layout, navigation, and content. Although their article did not describe the category under which this problem fell, it feels that none of these three would be a good match. Our ontology provides an alternative option, as this problem can be categorized under accessibility or guidance and support, depending on the specific formulation of the usability problem. Second, Mirkovic et al [54] evaluated the usability of an eHealth application that has two health goals: (1) patient-centered care and (2) self-management of a chronic illness. Their study found that users’ evaluation of the usefulness of system modules is based on the need for these modules within their phase of illness. Self-management modules were mostly useful for users who were recently diagnosed. For users who are in a more advanced phase of the illness, patient-doctor communication modules were more important. Although Mirkovic et al [54] categorized this problem as a useful problem, our ontology would suggest the category task-technology fit, as it illustrates how the health goals of a user depend on their phase of illness, which influences the users’ opinions on the usability of the evaluated system. Third, Stinson et al [55] found that users had difficulty understanding the labels of the classification of medication types. Although they classified this as a presentation error, our analysis revealed similar problems related to the understanding of medical information and terminology. In addition to problems related to the health context, Hattink et al [56] showed that experiencing technical problems is also a major reason for not using systems. Although it seems logical that system errors can affect user friendliness, many benchmarking instruments or heuristics [57,58] do not mention this aspect. In contrast, it was a frequent problem that was identified in our content analysis of usability problems.

With regard to the similarities between, on the one hand, our conceptualization of usability for eHealth and, on the other hand, usability questionnaires, such as the PSSUQ [8], SUS [10], Health-ITUES [11], and MAUQ [12], we observed that each questionnaire measures some of the usability factors we identified in our ontology. For example, the PSSUQ contains items on general system interaction, error management, interface organization, and procedural system information. The SUS contains items on general system interaction, interface organization, and structure. Both of these general usability questionnaires do not consider other general usability factors, such as technical performance, task-technology fit, design clarity, navigation, and health usability factors. eHealth usability benchmarking instruments, such as Health-ITUES and MAUQ, are more suited to measure how an eHealth application can support users in self-managing their health or be applied in a medical context. The Health-ITUES focuses on how the system fits to the daily clinical setting but neglects factors such as navigation, understandability of medical terminology, or interface organization. The MAUQ includes items on how a mobile health app supports users in managing their health and receiving health care or services, in addition to some general usability items such as navigation and interface organization. Each of these four questionnaires covered a handful of the usability factors identified in this study. Our ontology provides a more detailed and thorough overview of the most common usability factors that could hinder the usability of eHealth applications. Therefore, the currently available questionnaires are limited in their predictive value for determining the actual usability of an eHealth application.

### Limitations

This study had two main limitations. First, we intended to include data sets from a wide variety of eHealth application designed for different end-user groups. This was deemed necessary, as we wanted to develop a framework for eHealth applications in general. However, the eHealth applications that we included were, although quite diverse in nature, largely intended for middle-aged or older adults (aged ≥40 years). eHealth applications for other age groups, such as adolescents, could have specific usability problems that are underrepresented in this framework. Future research should determine if these other target groups have other common usability problems that need to be included in the eHealth usability ontology. Second, the Cohen κ values of the intercoder agreement were, although sufficient, not strong. One reason for the low Cohen κ scores is that usability problems were often ambiguously formulated. Although we excluded many of these problems beforehand, during coding it became notable that the researchers had different opinions about the origins of some problems. This is not completely avoidable in qualitative research but does highlight the common problem in usability evaluation studies: the evaluator effect [59]. The usability researcher has a large influence on the output of usability evaluation studies (and thus the formulation of usability problems). A means to establish a more uniform approach for formulating usability problems was provided by Khajouei et al [60]. It describes a framework for high-quality reporting of usability problems that mentions the underlying causes, severity, and impact on task performance. Furthermore, the use of a standardized framework for coding usability problems can provide support against the evaluator effect, as it helps create a common ground between researchers.

### Conclusions

The current set of usability benchmarking instruments only provides a limited overview of the usability of eHealth applications, as they do not consider eHealth-specific factors. Our reconceptualization of usability in the eHealth context will help practitioners and researchers better understand the usability problems they encounter in their evaluations and develop suitable benchmarking tools.

## Abbreviations

GUI: general user interface
Health-ITUES: Health Information Technology Usability Evaluation Scale
ISO: International Organization for Standardization
MAUQ: Mental Health App Usability Questionnaire
PSSUQ: Poststudy System Usability Questionnaire
SUS: system usability scale
